# Supplementary material for: Computational Design of Gas Sensors Based on V3S4 Monolayer
Source: Nanomaterials (Basel). 2022 Feb 25;12(5):774. doi: 10.3390/nano12050774 (PMC8912300; doi:10.3390/nano12050774)
Supplement: Supplementary file 1 [file nanomaterials-12-00774-s001.zip › nanomaterials-1603590-supplementary.pdf]

Supplementary Materials

Computational Design of Gas Sensors Based on V_3S_4 Monolayer

Ilya V. Chepkasov [1], Ekaterina V. Sukhanova [2], Alexander G. Kvashnin [1,*], Hayk A. Zakaryan [3], Misha A. Aghamalyan [3], Yevgeni Sh. Mamasakhlisov [4,5], Anton M. Manakhov [6], Zakhar I. Popov [2] and Dmitry G. Kvashnin [2]

1 Center for Energy Science and Technology, Skolkovo Institute of Science and Technology, Bolshoy Boulevard 30, bld. 1, 121205 Moscow, Russia; i.chepkasov@skoltech.ru
2 Emanuel Institute of Biochemical Physics RAS, 4 Kosygin Street, 119334 Moscow, Russia; yekaterina.sukhanova@phystech.edu (E.V.S.); zipcool@bk.ru (Z.I.P.); dgkvashnin@phystech.edu (D.G.K.)
3 Computational Materials Science Laboratory at the Center of Semiconductor Devices and Nanotechnology, Yerevan State University, 1 Alex Manoogian St., Yerevan 0025, Armenia; hayk.zakaryan@ysu.am (H.A.Z.); misha.aghamalyan@gmail.com (M.A.A.)
4 Department of Molecular Physics, Yerevan State University, 1 Alex Manoogian St., Yerevan 0025, Armenia;
y.mamasakhlisov@ysu.am
5 Department of Materials Technology and Structure of Electronic Technique, Russian-Armenian University, 123 Hovsep Emin St., Yerevan 0051, Armenia
6 Aramco Innovations LLC, Aramco Research Center, 119234 Moscow, Russia; anton.manakhov@aramcoinnovations.com
* Correspondence: a.kvashnin@skoltech.ru

SI. Crystal structure prediction

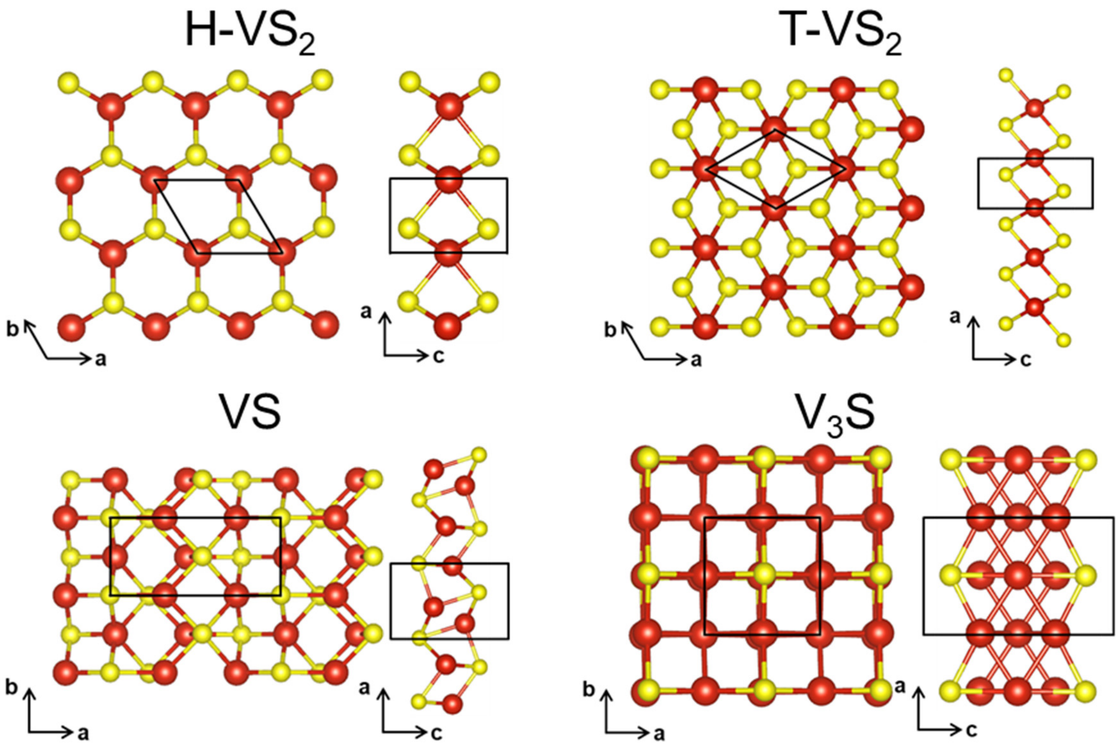

Figure S1. The atomic structures of predicted thermodynamically stable phases (except for V_3S_4).

Table S1. The lattice parameters of predicted V-S structures.

V-S phases	a, Å	b, Å	γ
H-VS_2	3.17	3.17	120
T-VS_2	3.18	3.18	120
VS	3.17	7.05	90
V_3S	4.22	4.22	90

SII. V_3S_4 phase

To prove the thermodynamic stability of the proposed V_3S_4 structure we decided to narrow down the search space and perform a fixed-composition evolutionary search for the most stable V_3S_4 structure with 14 atoms in the primitive cell, and after 25 generations V_3S_4 system was found to be similar to that we found in the variable-composition search.

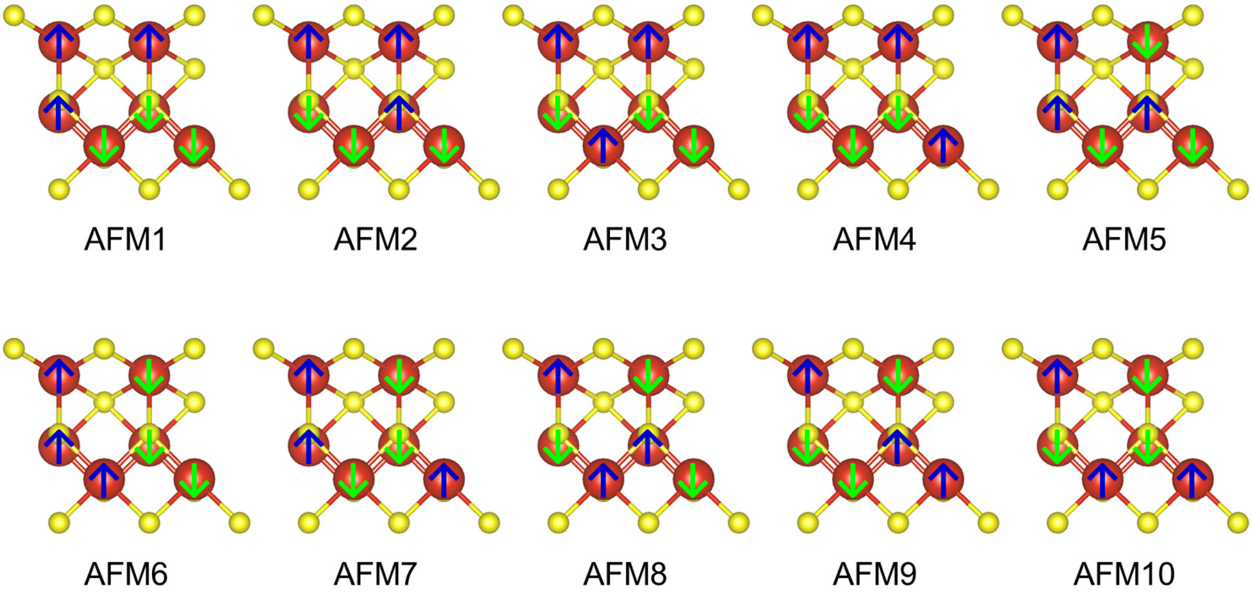

Figure S2. Considered antiferromagnetic configurations for 2 × 1 × 1 supercell of V_3S_4 phase.

Table S2. The energy difference between considered magnetic configurations and the most energetically favorable AFM10 configuration and absolute values of magnetic moment on vanadium atoms in square pyramidal (V_{sq_pyr}) and octahedral (V_{oct}) surroundings.

**Magnetic Configuration**	**ΔE, eV**	**V_{sq_pyr}, μ**	**V_{oct}, μ**
FM	0.29	2.28	2.64
AFM1	0.31	2.26	2.23
AFM2	0.31	2.26	2.23
AFM3	0.31	2.26	2.23
AFM4	0.31	2.26	2.23
AFM5	0.00	2.06	2.55
AFM6	0.09	2.15	2.52
AFM7	0.13	2.14	2.50
AFM8	0.13	2.14	2.50
AFM9	0.26	2.37	1.98
**AFM10**	**0.00**	**2.06**	**2.55**

SIII. Sensing properties

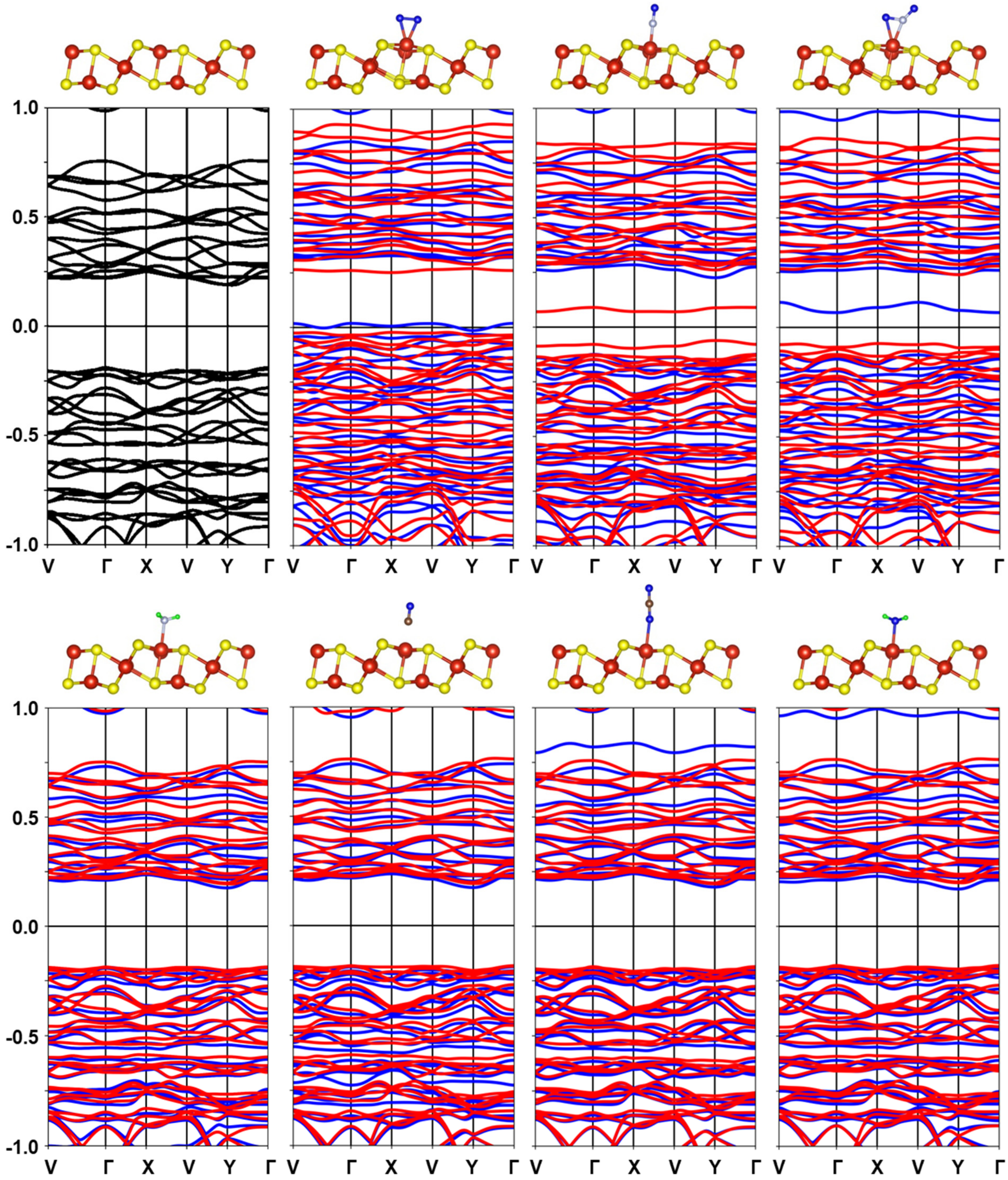

Figure S3. The most energetically favorable configuration of considered gas molecules (O_2, NO, NO_2, NH_3, CO, CO_2, and H_2O) on V_3S_4 surface and corresponding electronic band structures before and after the gas molecules adsorption. Spin Up and Down bands are shown by red and blue colors, respectively. By red, yellow, blue, brown, grey and green colors vanadium, sulfur, oxygen, carbon, nitrogen and hydrogen atoms are depicted.
